# miR-16-5p Regulates PTPN4 and Affects Cardiomyocyte Apoptosis and Autophagy Induced by Hypoxia/Reoxygenation

**DOI:** 10.1155/2021/5599031

**Published:** 2021-06-16

**Authors:** Zheng Cao, Jinglan Liu, Zhanqing Zhao, Qiao Wang

**Affiliations:** ^1^Emergency Department, Shanghai Ninth People's Hospital, School of Medicine, Shanghai Jiao Tong University, Shanghai 200011, China; ^2^International Peace Maternity and Child Health Hospital of China Welfare Institute, School of Medicine, Shanghai Jiao Tong University, Shanghai 200030, China; ^3^Department of Critical Care Medicine, Hainan Western Central Hospital, Danzhou 571799, Hainan Province, China

## Abstract

**Objectives:**

To explore the effects of miR-16-5p and PTPN4 on the apoptosis and autophagy of AC16 cardiomyocytes after hypoxia/reoxygenation treatment.

**Methods:**

AC16 cells were divided into the control group (NC), hypoxia/reoxygenation group (H/R), knockdown miR-16-5p negative control group (NC inhibitor), knockdown miR-16-5p group (miR-16-5p inhibitor), overexpression miR-16-5p negative control group (NC mimics), overexpression miR-16-5p group (miR-16-5p mimics), silent PTPN4 negative control group (sh-NC), silent PTPN4 group (sh-PTPN4), and silent PTPN4 + knockdown miR-16-5p group (sh-PTPN4 + miR-16-5p inhibitor). Real-time fluorescent quantitative PCR (RT-qPCR) and western blotting (WB) were used to measure the expression level of miR-16-3p, miR-16-5p, protein tyrosine phosphatase nonreceptor type 4 (PTPN4), and autophagy-related proteins (beclin-1, LC3 II/I, and P26) in AC16 cells. The apoptosis level of AC16 cells in each group was measured by flow cytometry and TUNEL. The dual-luciferase reporter gene experiment was also used to verify the targeting relationship between miR-16-5p and PTPN4.

**Results:**

After H/R treatment, the levels of myocardial injury markers including LDH and CK-MB in AC16 cells were increased significantly (*P* < 0.05), and the levels of cell apoptosis and autophagy also increased significantly (*P* < 0.05). The level of miR-16-3p in AC16 cells did not change significantly after H/R treatment, whereas the level of miR-16-5p was increased significantly (*P* < 0.05). After miR-16-5p was knocked down, the levels of LDH and CK-MB in AC16 cells treated with H/R were significantly reduced (*P* < 0.05), and the rates of cell apoptosis and autophagy were also significantly reduced (*P* < 0.05). miR-16-5p negatively regulated the expression level of PTPN4 protein in AC16 cells (*P* < 0.05), and the dual-luciferase reporter gene experiment confirmed that PTPN4 was the downstream target of miR-16-5p. Silencing of PTPN4 significantly increased the damage of AC16 cells induced by H/R treatment (*P* < 0.05), but simultaneously inhibiting the expression of PTPN4 and miR-16-5p reversed the protective effect of miR-16-5p knockdown on AC16 cells (*P* < 0.05).

**Conclusions:**

The expression of miR-16-5p is upregulated in AC16 cells after H/R treatment and the knockdown which can protect AC16 cells from H/R-induced cell damage that may be due to its regulation on the expression of PTPN4.

## 1. Introduction

Acute myocardial infarction is a common cardiovascular disease [1]. When it occurs, long-term ischemia and hypoxia can lead to interstitial fibrosis of myocardial cells [2], which in turn can induce left ventricular remodeling and heart failure. [3]. Coronary artery interventional therapy is currently the primary method for treating this disease [4], which can usually improve the cardiovascular function of patients with acute myocardial infarction after surgery. However, the prognosis of patients is still lacking [5]. Therefore, exploring the pathogenesis of acute myocardial infarction and looking for specific biomarkers is essential to improve patients' prognosis with acute myocardial infarction.

MicroRNA (miRNA) is a type of small noncoding RNA with a length of 18–22 nucleotides, which can bind with the 3′-untranslated region (UTR) of the downstream target gene to inhibit the expression of the target gene [6]. Previous studies have shown that the abnormal expression of various miRNAs is associated with acute myocardial infarction [7]. For example, it has been demonstrated that miR-15 can reduce the infarct size after acute myocardial infarction and enhance heart function [8]. miR-34a can significantly promote the senescence of cardiomyocytes. And the knockdown of miR-34a can reduce the apoptosis of cardiomyocytes and therefore reduce the area of myocardial infarction in model rats [9]. Previous studies have also shown that miR-16 has a particular role in promoting cardiovascular endothelial injury [10]. Marques et al. showed that miR-16-5p might be related to the occurrence of heart failure [11], and Hromadnikova et al. also suggested that miR-16-5p might also be associated with the occurrence of cardiovascular and cerebrovascular diseases in women [12]. In addition, the study by Wang et al. [13] showed that miR-16-5p could increase the viability of cardiomyocytes and promote the growth of vascular endothelial cells by targeting IRS1. At the same time, it can also inhibit the apoptosis of H/R cardiomyocytes, which is a potential therapeutic target for the treatment of myocardial infarction. The bioinformatics prediction results showed that Protein Tyrosine Phosphatase Nonreceptor Type 4 (PTPN4) is the potential downstream target gene of miR-16-5p. Studies have shown that PTPN4 can affect the biological behavior of cells through the activation of trypsin and calpain, which can affect the apoptosis of neuronal cells and regulate the homeostasis of neuronal cells. It has also been shown that silencing of PTPN4 can induce the apoptosis of glioblastoma [14], but fewer studies focus on the role of PTPN4 in myocardial infarction. Therefore, we constructed a hypoxia/reoxygenation (H/R) cell model in this study to explore the effects of miR-16-5p and PTPN4 on the apoptosis and autophagy AC16 cardiomyocytes after H/R treatment.

## 2. Materials and Methods

### 2.1. Cells and Main Reagents

Cells: AC16 human cardiomyocytes (PCS100-012) were purchased from ATCC (USA). Main reagents: fetal bovine serum (Cat. no. 10099-141) and ethanol (Cat. no. 64-17-5) were purchased from Shanghai Guchen Biotechnology Co., Ltd. TBST solution (number: SJH1021) and 5% skimmed milk powder (Cat. no. SJH1037) were purchased from Shanghai Ruji Biotechnology Development Co., Ltd., China. Penicillin/streptomycin solution (Cat. no. KL180120) and PBS buffer (Cat. no. KL180326) were purchased from Shanghai Kanglang Biotechnology Co., Ltd., China. Annexin V-FITC/PI Apoptosis Detection Kit (Cat. no. V13242), RIPA Lysis Solution (Cat. no. 89900), TRIzol Reagent (Cat. no. 15596018), BCA Protein Concentration Determination Kit (Cat. no. 23229), ECL Chemiluminescence Reagent Kit (Cat. no. 35050), Lipofectamine 2000 Kit (Cat. no. 11668019), PrimeScript RT Master Mix Kit (Cat. no. 11752050), SYBE Green Kit (Cat. no. A46112), Lactate Dehydrogenase (LDH) Detection Kit (Cat. no. EPX010-12262-901), and Creatine Kinase Isozyme (CK-MB) Detection Kit (Cat. no. AKC03005) were all purchased from Thermo Fisher Company, USA. Rabbit anti-human beclin-1 polyclonal antibody (Cat. no. ab62557), rabbit anti-human microtubule associated protein 1 light chain 3 beta (LC3B) polyclonal antibody (Cat. no. ab48394), rabbit anti-human *P*26 polyclonal antibody (Cat. no. ab223175), and rabbit anti-human horseradish peroxidase-labeled IgG antibody (Cat. no. ab6759) were purchased from Abcam, USA. Rabbit anti-human PTPN4 polyclonal antibody (Cat. no. IMG-5143A) and rabbit anti-human GAPDH polyclonal antibody (Cat. no. NBP1-80867) were purchased from Novus, USA. The dual-luciferase reporter gene detection kit (Cat. no. AC19L021) was purchased from Shanghai Liji Biotechnology Co., Ltd. The primers were designed and synthesized by Guangzhou Boxin Biotechnology Co., Ltd., China.

### 2.2. Cell Culture and Transfection

AC16 cells were cultured in DMEM medium containing 10% fetal bovine serum and 1% penicillin/streptomycin for the routine culture which was then seeded into a 6-well plate at a density of 2 × 10^5^ cells per well, and they were randomly divided into a blank control group (NC), a knockdown miR-16-5p negative control group (NC inhibitor), and a knockdown miR-16-5p group (miR-16-5p inhibitor), overexpression of miR-16-5p negative control group (NC mimics), overexpression of miR-16-5p group (miR-16-5p mimics), silent PTPN4 negative control group (sh-NC), silent PTPN4 group (sh-PTPN4), and silent PTPN4 + knockdown of miR-16-5p group (sh-PTPN4 + miR-16-5p inhibitor). Following the manufacturer's instructions, Lipofectamine 2000 kit was then used to transfect the corresponding vector into AC16 cells, which were then incubated for 24 h in a constant temperature incubator at 37°C with 5% CO_2_ for subsequent use.

### 2.3. Construction of the Hypoxia/Reoxygenation (H/R) Cell Model

We constructed a hypoxia/reoxygenation (H/R) cell model based on the method of Yu et al. [15]. The control group and the transfected AC16 cells of each group were incubated in a constant temperature incubator containing 5% CO_2_ and 95% N_2_ at 37°C for 4 h to simulate the ischemic environment of cardiomyocytes. After that, the cells were incubated for 3 h in a constant temperature incubator containing 5% CO_2_ and 95% O_2_ at 37°C. Each group of cells was then collected for subsequent experiments. Strictly following the instructions, the LDH detection kit and CK-MB detection kit were used to measure the levels of LDH and CK-MB in each group of cells and evaluate the degree of myocardial cell damage in each group.

### 2.4. Flow Cytometry Detects the Cell Apoptosis

AC16 cells in each group were collected, and the cells were washed twice with precooled PBS buffer for 3 min each time. The washed cells were then centrifuged (1000 r/min) for 5 min and fixed with precooled ethanol (70%). After fixation, cells were centrifuged (1000 r/min) for another 5 min to resuspend them in PBS buffer. A 400-mesh screen was then used to filter the cells. 100 *μ*L of Annexin V-FITC was then added to stain the cells at room temperature for 15 min, and then 5 *μ*L of PI staining solution was added for staining at 4°C for 30 min. Immediately after staining, the cells were put into the flow cytometry.

### 2.5. TUNEL Staining

The AC16 cell slides of each group were collected, dried, and fixed with precooled ethanol (75%). After washing twice with PBS, the cells were infiltrated into 0.1% Triton X/100 sodium citrate solution. Strictly follow the instructions of the TUNEL Apoptosis Detection Kit to stain the cells in each group and let them react for 1 h at 37°C while being protected from light. Then DAPI was added to stain the cells and the slides were taken out after 7 minutes, observed and photographed under a fluorescence microscope.

### 2.6. Western Blotting (WB)

After collecting each group of cells, RIPA lysate was used to extract the total protein in AC16 cells. The concentration of the extracted protein was determined according to the instructions of the BCA protein concentration determination kit. After SDS-PAGE electrophoresis, the protein sample was transferred to the PVDF membrane. After blocking with 5% skimmed milk powder for 1 h, rabbit anti-human beclin-1 polyclonal antibody (1 : 1000), rabbit anti-human LC3B polyclonal antibody (1 : 1000), rabbit anti-human *P*26 polyclonal antibody (1 : 1000), rabbit anti-human PTPN4 polyclonal antibody (1 : 1000), and rabbit anti-human GAPDH polyclonal antibody (1 : 1000) were added and incubated overnight at 4°C. After washing the membrane 3 times with TBST, the horseradish peroxidase-labeled secondary antibody (1 : 3000) was added and incubated at room temperature for 1 h. The bands were developed by following the instructions of the ECL chemiluminescence kit, and then the gray value of the band was analyzed using ImageJ software.

### 2.7. Real-Time Fluorescent Quantitative PCR (RT-qPCR)

After collecting AC16 cells in each group, TRIzol reagent was used to extract the total RNA in each group of cells, and the RNA was then reverse transcribed into cDNA by following the PrimeScript RT Master Mix Kit instructions. U6 was then used as an internal reference to measure the expression levels of miR-16-3p and miR-16-5p in cells by following the SYBR Green kit instructions. The primer sequences used in this study are shown in Table 1. The reaction system contained 2 *μ*L reverse transcription product, 10 *μ*L SYBR Green Mix reagent, 0.5 *μ*L (10 *μ*mol/L) of forward and reverse primers, and 7 *μ*L of dH_2_O. The reaction conditions for a total of 45 cycles were set as follows: 95°C predenaturation for 5 min, denaturation at 94°C for 30 s, and annealing at 60°C for 30 s. The relative expression levels of miR-16-3p and miR-16-5p were calculated by the 2^−△△Ct^ method.

### 2.8. Dual-Luciferase Reporter Gene Experiment

The wild type PTPN4 (WT-PTPN4) sequence containing miR-16-5p 3′-UTR and the mutant PTPN4 (MUT-PTPN4) sequence without miR-16-5p 3′-UTR were inserted into the dual-luciferase report vectors. The dual-luciferase reporter vectors, NC mimics, and miR-16-5p mimics were then cotransfected into AC16 cells, and the cells were incubated at 37°C with 5% CO_2_ for 48 h. The intracellular luciferase activity of each group was determined according to the instructions of the dual-luciferase reporter gene detection kit.

### 2.9. Statistical Analysis

All experiments in this study were repeated 3 times and SPSS 22.0 was used to analyze the data. The data were expressed as the mean ± standard deviation, and the *t*-test was used to compare the difference between the two groups. *P* < 0.05 was used to indicate that the difference was statistically significant.

## 3. Results

### 3.1. Construction of the Hypoxia/Reoxygenation Cell Model

**Figure 1:**
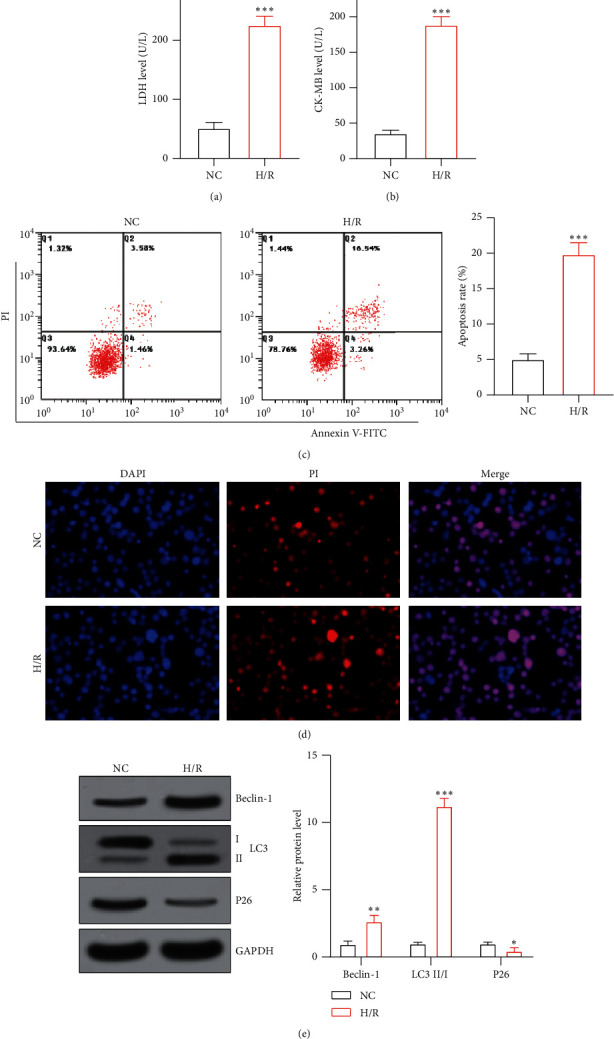
H/R-induces AC16 cell apoptosis and autophagy: (a) LDH levels in AC16 cells; (b) CK-MB levels in AC16 cells; (c) AC16 cell apoptosis level; (d) AC16 cell apoptosis detected by TUNEL fluorescence staining; (e) autophagy-related protein levels in AC16 cells. ^*∗*^*P* < 0.05, ^*∗*^*P* < 0.01, and ^*∗*^*P* < 0.001 vs. the NC group.

After AC16 cells were treated with H/R, the levels of LDH and CK-MB in the cells increased significantly (*P* < 0.05), as shown in Figures 1(a) and 1(b). The results of flow cytometry and TUNEL showed that the apoptosis rate of AC16 cells was significantly increased after H/R treatment (*P* < 0.05), as shown in Figures 1(c) and 1(d). WB results showed that the expression levels of beclin-1 and LC3 II/I protein in AC16 cells were significantly increased after H/R treatment (*P* < 0.05), whereas the *P*26 protein level was significantly reduced (*P* < 0.05); that is, H/R treatment can increase the level of autophagy in AC16 cells, as shown in Figure 1(e).

### 3.2. Knockdown of miR-16-5p Can Inhibit AC16 Cell Apoptosis and Autophagy Induced by Hypoxia/Reoxygenation

**Figure 2:**
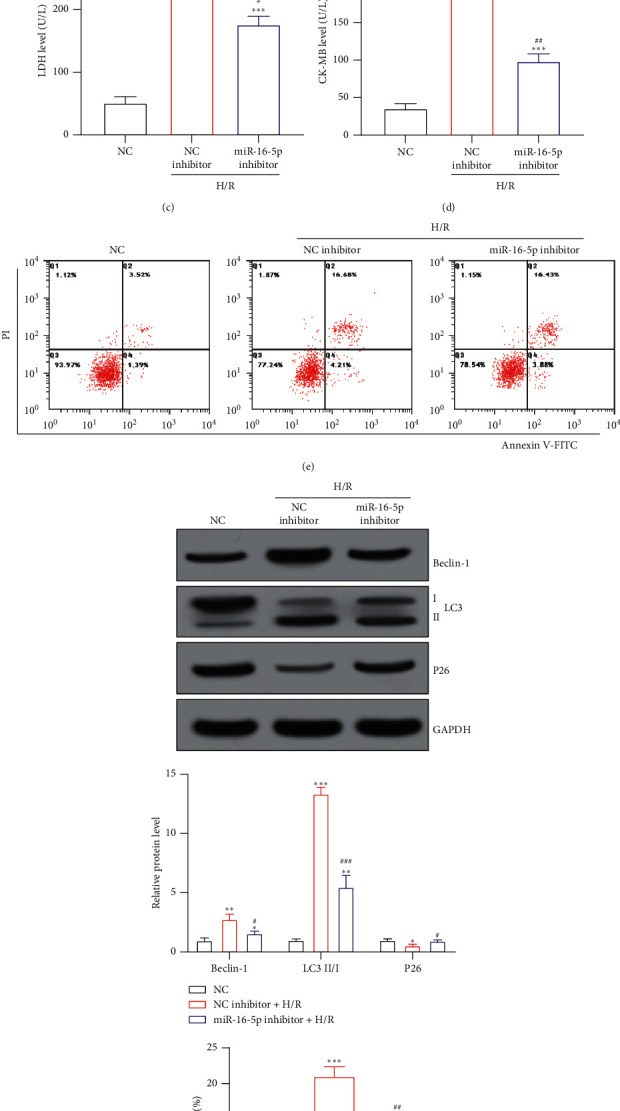
Knockdown of miR-16-5p inhibits H/R-induced apoptosis and autophagy in AC16 cells: (a, b) miR-16 levels in AC16 cells; (c) LDH levels in AC16 cells; (d) CK-MB levels in AC16 cells; (e) AC16 cell apoptosis level; (f) autophagy-related protein levels in AC16 cells. ^*∗*^*P* < 0.05, ^*∗∗*^*P* < 0.01, and ^*∗∗∗*^*P* < 0.001 vs. the NC group; ^#^*P* < 0.05, ^##^*P* < 0.01, and ^###^*P* < 0.001 vs. the NC inhibitor + H/R group.

The expression level of miR-16 in AC16 cells was measured by RT-qPCR. The results showed that the expression level of miR-16-3p in AC16 cells did not change significantly after H/R treatment (*P* < 0.05), while the expression level of miR-16-5p was a significant increase (*P* < 0.05), as shown in Figure 2(a). After knocking down miR-16-5p, the expression level of miR-16-5p in AC16 cells was significantly reduced (*P* < 0.05), as shown in Figure 2(b). After AC16 cells with miR-16-5p knocked down were treated with H/R, the levels of LDH and CK-MB in the cells were significantly decreased (), as shown in Figures 2(c) and 2(d). Finally, the results of flow cytometry and WB showed that knocking down miR-16-5p significantly inhibited the increase of AC16 cell apoptosis and autophagy induced by H/R treatment (*P* < 0.05), as shown in Figures 2(e) and 2(f).

### 3.3. PTPN4 Is the Downstream Target of miR-16-5p

**Figure 3:**
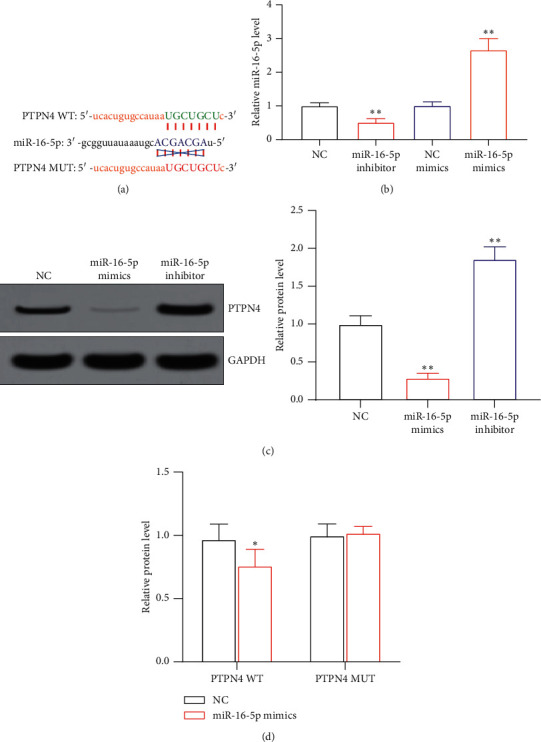
PTPN4 is a downstream target gene of miR-16-5p: (a) miR-16-5p and PTPN4 target binding sequence; (b) miR-16-5p levels in AC16 cells; (c) PTPN4 protein levels in AC16 cells; (d) luciferase activity in AC16 cells. ^*∗*^*P* < 0.05 and ^*∗∗*^*P* < 0.05 vs. the NC group.

The prediction results of StarBase showed that PTPN4 is the downstream target gene of miR-16-5p, and its target binding sequence is shown in Figure 3(a). After overexpression of miR-16-5p, the expression level of miR-16-5p in AC16 cells was significantly increased (*P* < 0.05), and its expression level was significantly decreased after its knockdown (*P* < 0.05), as shown in Figure 3(b). WB results showed that overexpression of miR-16-5p significantly inhibited the expression level of PTPN4 protein in AC16 cells (*P* < 0.05) while knocking down miR-16-5p can significantly increase the expression of PTPN4 (*P* < 0.05), as shown in Figure 3(c). The results of the dual-luciferase reporter gene experiment showed that overexpression of miR-16-5p significantly inhibited the luciferase activity of WT-PTPN4 (*P* < 0.05) but had no significant effect on the luciferase activity of MUT-PTPN4, as shown in Figure 3(d).

### 3.4. Silencing PTPN4 Reverses the Inhibitory Effect of H/R-Induced AC16 Cell Damage Caused by miR-16-5p Knockdown

**Figure 4:**
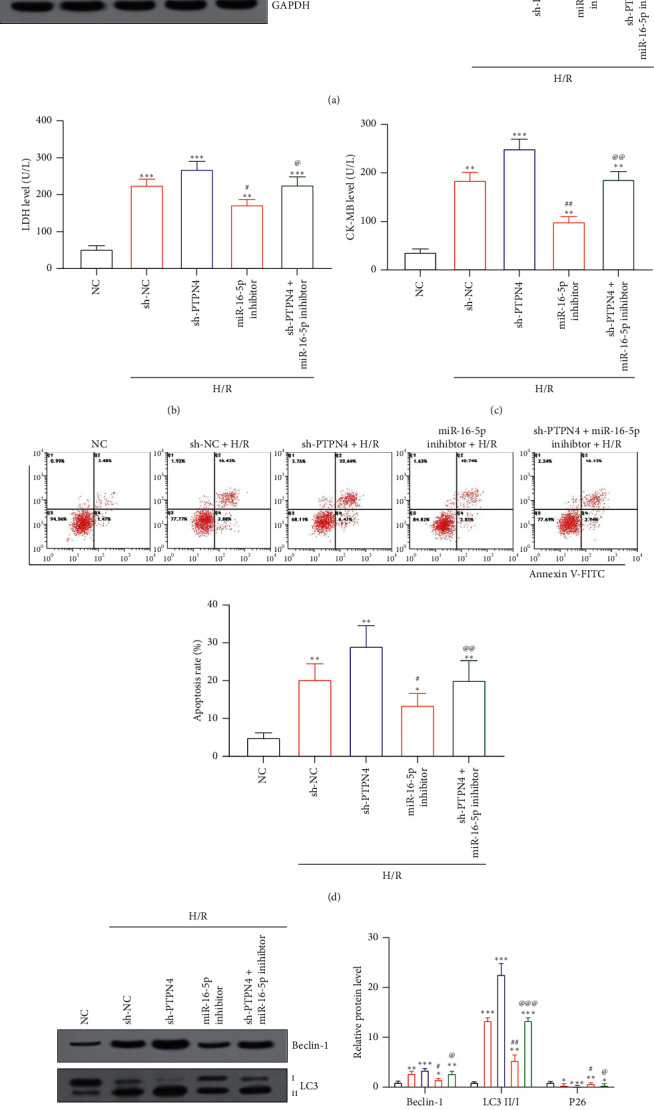
Silencing PTPN4 reverses the inhibitory effect of knockdown of miR-16-5p on H/R-induced AC16 cell damage: (a) PYPN4 protein in AC16 cells; (b) LDH levels in AC16 cells; (c) CK-MB levels in AC16 cells; (d) AC16 cell apoptosis level; (e) autophagy-related protein levels in AC16 cells. ^*∗*^*P* < 0.05,  ^*∗*^ ^*∗*^*P* < 0.01, and  ^*∗*^ ^*∗*^ ^*∗*^*P* < 0.001 vs. the NC group; ^#^*P* < 0.05 and ^##^*P* < 0.01 vs. the sh-NC + H/R group; ^@^*P* < 0.05, ^@@^*P* < 0.01, and ^@@@^*P* < 0.001 vs. the miR-16-5p inhibitor + H/R group.

WB results showed that H/R treatment significantly inhibited the expression of PTPN4 protein in AC16 cells (*P* < 0.05). After silencing PTPN4, the expression level of PTPN4 protein in cells was further reduced (*P* < 0.05). Knockdown of miR-16-5p on the other hand can upregulate the expression of PTPN4 protein (*P* < 0.05), which however can be reversed by the silencing of PTPN4 (*P* < 0.05), as shown in Figure 4(a). After PTPN4 silenced AC16 cells were treated with H/R, the levels of LDH and CK-MB in the cells were significantly increased (*P* < 0.05). Knockdown of miR-16-5p can significantly inhibit the increase in LDH and CK-MB levels in AC16 cells induced by H/R treatment (*P* < 0.05), but PTPN4 silencing reversed this inhibition (*P* < 0.05) as shown in Figures 4(b) and 4(c). The results of flow cytometry and WB showed that the levels of apoptosis and autophagy of AC16 cells with silenced PTPN4 were significantly increased after H/R treatment (*P* < 0.05). Whereas the knockdown of miR-16-5p can significantly inhibit the increase of AC16 cell apoptosis and autophagy induced by H/R treatment (*P* < 0.05), the silence of PTPN4 can effectively reverse this protective effect of knockdown miR-16-5p on AC16 cells (*P* < 0.05) as shown in Figures 4(d) and 4(e).

## 4. Discussion

Cardiomyocytes are prone to apoptosis and autophagy after H/R can cause heart tissue damage, resulting in a decrease in heart volume, and can affect the contractile function of the heart [16]. Therefore, exploring the mechanism to alleviate H/R-induced cardiomyocyte apoptosis and autophagy is clinically important. In this study, it was found that the levels of myocardial injury markers including LDH and CK-MB in AC16 cells were significantly increased after H/R treatment, and the levels of apoptosis and autophagy were also significantly increased. Previous studies have shown that miRNA is involved in the occurrence and development of acute myocardial infarction. Liang et al. showed that the expression level of miR-122 in H9C2 cells after H/R treatment was upregulated, and after targeted binding with GATA-4, it inhibited the expression of GATA-4, thereby promoting cardiomyocyte apoptosis [17]. Liu et al. showed that inhibiting miR-101-3*p* can inhibit the activation of the JAK2/STAT3 signaling pathway and downregulate the apoptosis of cardiomyocytes after H/R treatment [18]. And Hu et al. showed that miR-138-5*p* was downregulated in H9C2 cells after H/R treatment and miR-138-5*p* can promote H/R-induced cardiomyocyte apoptosis and inflammation in vitro [19]. miR-16-5p is a miRNA located on human chromosome 3, which plays an important role in the process of cardiomyocyte apoptosis and cardiovascular formation [13]. In this study, we found that the expression level of miR-16-5p in AC16 cells after H/R treatment was significantly increased. One the other hand, after inhibiting the expression of miR-16-5p, the levels of LDH and CK-MB in AC16 cells treated with H/R were decreased, and their apoptosis and autophagy levels were also significantly reduced, suggesting that inhibiting the expression of miR-16-5p can reduce AC16 cell damage caused by H/R.

PTPN4 is a nontyrosine phosphatase receptor, which can unbalance the interaction between TRAM and TRIP by inhibiting tyrosine phosphorylation and cytoplasmic heterotopy of TRAM and specifically inhibit the activation of TLR4 and its downstream NF-*κβ*, thereby regulating the apoptosis and autophagy of cardiomyocytes [20]. Ge et al. showed that knocking down the expression of PTPN4 can aggravate the H/R-induced H9C2 cardiomyocyte damage and can increase the H/R-induced apoptosis of H9C2 cells [21]. Bioinformatic prediction showed that PTPN4 is a downstream target gene of miR-16-5p, and overexpression of miR-16-5p significantly inhibited the expression of PTPN4 in AC16 cells. Besides, after reducing the expression of PTPN4, the H/R-induced AC16 cell damage was aggravated, and the levels of apoptosis and autophagy were also increased significantly. These results suggest that there is a negative feedback regulation between miR-15-5*p* and PTPN4, and the expression level of PTPN4 is closely related to the degree of AC16 cell damage induced by H/R.

PTPN4 can regulate the activity of the TKR4/NK-*κβ* signaling pathway, thereby affecting the damage of cardiomyocytes. Based on the results of this study, we speculate that miR-16-5p can affect the degree of H/R-induced cardiomyocyte damage by targeting PTPN4 and thereby regulating the activity of the TKR4/NK-*κβ* pathway. However, given the experimental conditions and funding limitations, this study did not verify the above-mentioned pathways, and that needs to be explored in the follow-up study. In conclusion, the results of this study confirmed that miR-16-5p was upregulated in AC16 cells induced by H/R and promoted AC16 cardiomyocyte apoptosis and autophagy by inhibition of PTPN4 expression.

## Figures and Tables

**Table 1:** Primer sequences.

Gene	Sequence
miR-16-3p	Forward: 5′-TTTTTAAAATAGGAAGGGGATGATT-3′
	Reverse: 5′-TCCCTATCACACTAAAACAACACAA-3′

miR-16-5p	Forward: 5′-TAGCAGCACGTAAATATTGGCG-3′
	Reverse: 5′-TGCGTGTCGTGGAGTC-3′

U6	Forward: 5′-CTCGCTTCGGCAGCACATA-3′
	Reverse: 5′-AACGCTTCACGAATTTGCGT-3′

## Data Availability

The data and materials used to support the findings of this study are included within the published article.

## References

[1] Reed G. W., Rossi J. E., Cannon C. P. (2017). Acute myocardial infarction. *The Lancet*.

[2] Yin Y., Zhang Q., Zhao Q. (2019). Tongxinluo attenuates myocardiac fibrosis after acute myocardial infarction in rats via inhibition of endothelial-to-mesenchymal transition. *BioMed Research International*.

[3] Anzai T. (2018). Inflammatory mechanisms of cardiovascular remodeling. *Circulation Journal*.

[4] Hausenloy D. J., Chilian W., Crea F. (2019). The coronary circulation in acute myocardial ischaemia/reperfusion injury: a target for cardioprotection. *Cardiovascular Research*.

[5] Shah A. H., Puri R., Kalra A. (2019). Management of cardiogenic shock complicating acute myocardial infarction: a review. *Clinical Cardiology*.

[6] Tiwari A., Mukherjee B., Dixit M. (2018). MicroRNA key to angiogenesis regulation: MiRNA biology and therapy. *Current Cancer Drug Targets*.

[7] Chistiakov D. A., Orekhov A. N., Bobryshev Y. V. (2016). Cardiac-specific miRNA in cardiogenesis, heart function, and cardiac pathology (with focus on myocardial infarction). *Journal of Molecular and Cellular Cardiology*.

[8] Hullinger T. G., Montgomery R. L., Seto A. G. (2012). Inhibition of miR-15 protects against cardiac ischemic injury. *Circulation Research*.

[9] Boon R. A., Iekushi K., Lechner S. (2013). MicroRNA-34a regulates cardiac ageing and function. *Nature*.

[10] Sun C.-Y., She X.-M., Qin Y. (2013). miR-15a and miR-16 affect the angiogenesis of multiple myeloma by targeting VEGF. *Carcinogenesis*.

[11] Marques F. Z., Vizi D., Khammy O., Mariani J. A., Kaye D. M. (2016). The transcardiac gradient of cardio-microRNAs in the failing heart. *European Journal of Heart Failure*.

[12] Hromadnikova I., Kotlabova K., Dvorakova L., Krofta L. (2020). Evaluation of vascular endothelial function in young and middle-aged women with respect to a history of pregnancy, pregnancy-related complications, classical cardiovascular risk factors, and epigenetics. *International Journal of Molecular Sciences*.

[13] Wang X., Shang Y., Dai S., Wu W., Yi F., Cheng L. (2020). MicroRNA-16-5*p* aggravates myocardial infarction injury by targeting the expression of insulin receptor substrates 1 and mediating myocardial apoptosis and angiogenesis. *Current Neurovascular Research*.

[14] Caillet-Saguy C., Toto A., Guerois R. (2017). Regulation of the human phosphatase PTPN4 by the inter-domain linker connecting the PDZ and the phosphatase domains. *Scientific Reports*.

[15] Yu Z., Ren Q., Yu S., Gao X. (2020). Sevoflurane protects cardiomyocytes against hypoxia/reperfusion injury via LINC01133/miR-30a-5*p* axis. *Bioscience Reports*.

[16] Wang K., Yuan Y., Liu X. (2016). Cardiac specific overexpression of mitochondrial omi/HtrA2 induces myocardial apoptosis and cardiac dysfunction. *Scientific Reports*.

[17] Liang W., Guo J., Li J., Bai C., Dong Y. (2016). Downregulation of miR-122 attenuates hypoxia/reoxygenation (H/R)-induced myocardial cell apoptosis by upregulating GATA-4. *Biochemical and Biophysical Research Communications*.

[18] Liu J., Wang J., Ning Y., Chen F. (2020). The inhibition of miR-101a-3*p* alleviates H/R injury in H9C2 cells by regulating the JAK2/STAT3 pathway. *Molecular Medcine Reports*.

[19] Hu X., Ma R., Cao J. (2020). CircSAMD4A aggravates H/R-induced cardiomyocyte apoptosis and inflammatory response by sponging miR-138-5*p*. *Journal of Cellular and Molecular Medicine*.

[20] Huai W., Song H., Wang L. (2015). Phosphatase PTPN4 preferentially inhibits TRIF-dependent TLR4 pathway by dephosphorylating TRAM. *The Journal of Immunology*.

[21] Ge L., Cai Y., Ying F. (2019). miR-181c-5*p* exacerbates hypoxia/reoxygenation-induced cardiomyocyte apoptosis via targeting PTPN4. *Oxidative Medicine and Cellular Longevity*.

